# EXPLORING LINGUISTIC PATTERNS ON A STORY RECALL TASK IN PEOPLE WITH MILD COGNITIVE IMPAIRMENT

**DOI:** 10.1093/geroni/igac059.2066

**Published:** 2022-12-20

**Authors:** Angela Boland, Adelaide Jensen, Patrick Davidson, Vanessa Taler

**Affiliations:** University of Ottawa, Ottawa, Ontario, Canada; University of Ottawa, Ottawa, Ontario, Canada; University of Ottawa, Ottawa, Ontario, Canada; University of Ottawa, Ottawa, Ontario, Canada

## Abstract

Mild cognitive impairment (MCI) involves declines in language and episodic memory. Episodic memory is often assessed using language tasks. To prevent linguistic factors from confounding recall scores, memory and language should be jointly examined. We explored linguistic patterns on a story recall task among cognitively healthy adults aged 65+ (n=18) and people with amnestic MCI (n=18). Participants completed immediate and delayed (20-30min) recall on a set of novel story recall materials (6 pairs, i.e., 12 stories total). Stories were coded using a propositional coding scheme (where a proposition refers to the smallest unit of meaning), as well as a unit scoring scheme (i.e., individual words). Responses were coded as veridical (word-for-word), gist (general idea), and distortion (error). Linguistic features of the output were coded using the Linguistic Inquiry and Word Count (LIWC) program. Overall, people with MCI produced more verbs, fewer time-related words, and fewer total words than control participants. In the MCI group, delayed unit- and proposition-based veridical and gist recall scores were positively correlated with certainty and causation words, indicating that higher certainty about events and their causal links is associated with better memory. Total words were positively correlated with all immediate and delayed recall scores, indicating that amount of linguistic output is strongly linked to memory in MCI. Time-related words were positively correlated with immediate unit-based veridical recall, suggesting that, in MCI, more words denoting time signal better immediate recall of story details. Examining linguistic features of verbal output in memory tasks could improve detection of MCI.

